# Dietary Glutamic Acid, Obesity, and Depressive Symptoms in Patients With Schizophrenia

**DOI:** 10.3389/fpsyt.2020.620097

**Published:** 2021-01-21

**Authors:** Pooja Kumar, A. Zarina Kraal, Andreas M. Prawdzik, Allison E. Ringold, Vicki Ellingrod

**Affiliations:** ^1^College of Pharmacy, University of Michigan, Ann Arbor, MI, United States; ^2^Ohio State University Wexner Medical Center, Columbus, OH, United States; ^3^Department of Psychiatry, School of Medicine, Medical University of South Carolina, Charleston, SC, United States; ^4^Department of Mathematics, Central Michigan University, Mount Pleasant, MI, United States; ^5^College of Wooster, Wooster, OH, United States; ^6^Department of Psychiatry, School of Medicine, University of Michigan, Ann Arbor, MI, United States

**Keywords:** glutamate, diet, depression, schizophrenia, obesity

## Abstract

**Introduction:** Schizophrenia is a lifelong condition associated with several comorbid conditions such as physical illnesses like obesity, as well as co-occurring psychiatric symptoms such as depression. Research regarding susceptibility to some of these comorbidities has primary focused on genetic risks or neurotransmitters and very little work has been done to understand environmental factors such as diet. In particular, understanding the role of dietary glutamic acid consumption on co-morbidities in patients with schizophrenia is important, as evidence suggests that glutamic acid consumption may directly influence glutamatergic neurotransmission; a key neurotransmitter related to schizophrenia, its associated co-morbidities, and depression. Therefore, the aim of this study was to examine the potential relationship between dietary glutamic acid and depressive symptomatology in patients with schizophrenia, stratified by obesity status, due to its relationship with inflammation, antipsychotic use, and depressive symptoms.

**Methods:** Subjects included in this analysis, were part of a parent cross-sectional study in which included three dietary recalls analyzed using protocols outlined as part of the National Health and Nutrition Examination Surveys (NHANES) standardized criteria. Additionally, body mass index (BMI), and Beck Depression Inventory were obtained at this visit. Subjects with a BMI ≥ 30 kg/m^2^ were included in the obesity group, and the relationship between glutamic acid consumption and BDI scores was analyzed after controlling for age, race, sex, antidepressant and antipsychotic use, and animal and vegetable protein intake which provide natural forms of dietary glutamic acid.

**Results:** A total of 168 participants were included in this study, of which 42.5% were female and 52.9% were White. The mean BMI for the group as a whole was 33.5 ± 8.7 (kg/m^2^) and the mean BDI was 14.5 ± 10.2 (range 2–50). No differences were found between obesity groups, other than a greater hyperlipidemia, hypertension, and lower waist to hip ratio. Overall, no relationship was found between dietary glutamic acid and BDI scores, However, for non-obese participants, diets higher levels of glutamic acid were associated with greater depression symptomatology (*p* = 0.021).

**Conclusion:** These preliminary results indicate a possible correlation between dietary glutamic acid a depressive symptoms in non-obese patients with schizophrenia, although further research is needed to specifically examine this relationship.

## Introduction

For those diagnosed with a serious mental illness, such as schizophrenia, co-morbid psychiatric and physical illnesses are commonplace. In fact, the prevalence of depressive symptoms within this patient population is ~40%, whereas the prevalence of obesity contributing to cardiovascular disease is upwards of 50% (1, 2). Why these conditions commonly co-occur is not fully understood, however genetic risk factors as well as common neurotransmitter pathways, have been identified (3, 4). Recently, research has focused on the role of glutamate, which acts primarily thorough the N-Methyl-D-aspartate (NMDA) receptor. Data shows that over activation of the NDMA receptors by glutamate can be neurotoxic and result in cell death (5). Additionally antipsychotics, like olanzapine and clozapine, used for the treatment of psychotic symptoms seen in persons with schizophrenia, can attenuate hyperglutamateric states resulting in their therapeutic effect, while also contributing to the occurrence of obesity and cardiovascular disease (6, 7). For depression, glutamate is also a neurotransmitter of interest, however its role is not as clearly understood (8, 9). Most work regarding glutamate's role has come from data showing that ketamine, a NMDA acting medication, is effective in the treatment of refractory depression, however, the mechanisms this therapeutic effect is not fully known (8).

In addition to glutamate neurotransmission, epigenomic relationships between obesity, cardiovascular disease, psychosis, and depressive symptoms have been reported (10). In particular, hypermethylation of genes common to known obesity and depression pathways [i.e., Brain Derived Neurotropic Factor (BDNF)] may trigger various inflammatory cascades, which link diet, obesity, and depressive symptoms (10–12). This work also supports findings reported by our group examining gene specific methylation differences between schizophrenia and cardiovascular risk factors (13–15). Thus, taken as a whole, the field needs to better understand the impact of environmental factors in patients with psychosis, as work regarding diet in schizophrenia spectrum disorder, and the occurrence of co-morbid disorders such as obesity, cardiovascular disease, and depressive symptoms is fairly limited (16–18).

The newly emerging field of nutritional psychiatry may provide some answers regarding this relationship as groups, such as ours, begin to examine the relationships between diet, obesity, and depression symptomatology. Key to this investigation is work that has focused on the role of dietary monosodium glutamate (MSG) in relation to psychiatric symptoms, pain response, and obesity. Emerging evidence suggests that MSG may directly influence glutamatergic neurotransmission, which underlies the pathophysiology of mental illnesses, including schizophrenia, and depressive disorders (8).

Chemically, glutamic acid is an amino acid necessary for the biosynthesis of glutamate, a key neurotransmitter. In a healthy diet, most glutamic acid is obtained through the consumption of meats, poultry, fish, eggs, and dairy products, as well as a few high protein vegetable sources. Monosodium glutamate (MSG) is the mono sodium salt form of glutamic acid, which is used as a food additive in commercially processed foods (19). Physiologically, both animal, and vegetable protein sources of glutamic acid, as well as the intake of MSG through processed foods, are key compounds used for the biosynthesis of glutamate. Glutamate is necessary for the functionality of key neurotransmitters and the body cannot distinguish between glutamic acid originating from animal and/or vegetable sources or MSG sources from processed foods. Thus, for individuals with diets high in processed foods, high MSG consumption may result in an abundance of glutamic acid, leading to hyperglutamatergic neurotransmission possibly contributing to psychiatric symptoms such as depressive symptoms (20).

Most research regarding the impact of MSG on mental health symptomatology comes from *in vitro* animal studies that have demonstrated a link between MSG consumption and the occurrence of anxiety and depression symptoms, as well as the occurrence of pain (21, 22). Briefly, rats exposed to MSG during early life are more likely to have behavioral patterns in line with animal models of depression and anxiety. Furthermore, limited data suggests that MSG administration in these rats leads to changes in brain morphology as seen by increased microglial cell density in the motor cortex, which occurred in a dose dependent manner (23). The authors hypothesized that these findings were indicative of hyperglutamateric neurotoxicity secondary to MSG administration.

In addition, MSG consumption is also linked to the occurrence of obesity, as studies have shown that rats exposed to MSG through subcutaneous injection during the first 10 days of life developed 20–42% higher body weights when compared to the control group (24). To add to this evidence, MSG has been used to induce obesity and diabetes in animals for further study (25). For human studies however, conflicting relationships have been observed between MSG consumption, body weight, and obesity (19, 26). Thus, further studies need to be done, especially given that other groups have reported preliminary relationships between MSG consumption and pain (27, 28), in patients diagnosed with myofascial temporomandibular disorders or fibromyalgia. Additionally, other groups have found that in healthy individuals, MSG consumption was also associated with 6-fold greater incidence of pain symptoms when compared to ingestion of an equivalent amount of sodium chloride (27–29).

Therefore, overall very little is known regarding dietary MSG and the occurrence of psychiatric symptoms in humans, and how obesity may alter patterns of association. Thus, with this in mind, the primary purpose of this investigation was to evaluate the relationship between dietary glutamic acid consumption, and depressive symptoms in obese and non-obese patients with psychotic disorders. We hypothesized that overall, there would be a positive association between dietary glutamic acid consumption and increasing depressive symptoms for schizophrenia subjects, and that this relationship would vary by obesity status.

## Methods

### Participants

Study participants included in this investigation, were part of a larger cross-sectional study that has been described elsewhere (30). Briefly subjects were included if they met the following inclusion criteria (i) aged 18–90 years; (ii) had a diagnosis of schizophrenia, schizophreniform disorder, schizoaffective disorder, psychosis not otherwise specified (NOS), using the Diagnostic and Statistical Manual of Mental Disorders, 4th edition (DSM-IV) criteria; and (iii) had been treated with an antipsychotic for 6 months. Participants were excluded if they were (i) unable to give informed consent or unwilling to participate; (ii) had an active substance dependence diagnosis; or (iii) had a documented history of type 2 Diabetes Mellitus prior to the antipsychotic use. Participants were recruited from outpatient clinics in the Southeastern Michigan region. The study was approved by the University of Michigan Medical School Institutional Review Board, the Washtenaw County Health Organization, the Ann Arbor Veterans Affairs Medical Center, and the Detroit Wayne County Community Mental Health Agency. The following assessments were collected as part of the parent study, and were used in this analysis.

### Assessments

All participants included in this analysis, provided written informed consent at their first study visit followed by a clinical interview, which included a psychiatric diagnostic assessment using the structured clinical interview for DSM-IV (SCID) (31). The Beck Depression Inventory (BDI) was used as the primary assessment for depressive symptomatology as this scale has demonstrated to be a reliable and valid measure of depressive symptoms in patients with schizophrenia (32). Subjects also answered questions regarding current and past medications, as well as current and previous medical history, which included information regarding comorbid conditions (i.e., hypertension, diabetes, and hyperlipidemia). These data were confirmed through review of each subjects' electronic health record. To standardize antipsychotic use/exposure, chlorpromazine (CPZ) equivalents were calculated for every participant using standardized methods (33, 34). Subjects also underwent a brief physical assessment which included height, weight, and hip and waist circumference. Both the height and weight measurements were used to calculate Body Mass Index (BMI). Subjects with a BMI ≥ 30 kg/m^2^ were considered obese while those with a BMI below this threshold were placed in the non-obese group.

Additionally for the parent study, subjects were asked to complete a 24 h food recall, conducted by the study's registered dietician, where each subject was guided in recalling all foods eaten within the 24 h before their study visit. This assessment was then repeated twice by phone on random occasions within 10 days after the in-person study visit, for a total of three recalls assessments. The dietary staff used standard protocols outlined as part of the National Health and Nutrition Examination Surveys (NHANES) training and used standard measuring guides to help the patients remember the volume and dimensions of food they consumed in the previous 24 h. Nutritional data from this information was calculated using the Nutrition Data Systems for Research (NDSR) software developed by the Nutrition Coordinating Center (NCC) at the University of Minnesota (35). For the current analysis, data were averaged across the three dietary recall occasions in terms of nutrient values. As the nutritional output from this assessment does not specifically measure MSG content, dietary intake related to glutamic acid, was used as a surrogate measure of MSG consumption, controlling for animal and vegetable protein intake, in each analysis, in order to determine excessive glutamic acid consumption.

### Statistical Analysis

Analyses were done using SPSS Statistics version 23 to analyze the relationship between dietary intake of glutamic acid and BDI scores in all subjects, as well as subjects stratified by obesity status (BMI ≥ 30 kg/m^2^; 24). Differences in baseline subject characteristics between the obese and non—obese groups were conducted using a chi—squared analysis to compare categorical variables and a *t-*test for continuous variables. A hierarchical regression tested the independent association between dietary intake of the glutamic acid, and depression symptoms assessed using the BDI, controlling for age, sex, race, antidepressant usage, CPZ equivalents, as well as animal and vegetable protein intake.

## Results

The study sample included 168 patients with baseline characteristics described in Table 1. Overall, the sample was 45.2% female and primarily White (52.9%). The mean BMI for the group as a whole was 33.5 ± 8.7 (kg/m^2^) and the mean BDI 14.5 ± 10.2, indicating mild depressive disturbances, however the range was 2–50. In terms of demographic differences between the obese and non-obese groups, on average, the obese group was older, had a higher proportion of women, included more White subjects, and had higher rates of hypertension and hyperlipidemia, and lower waist to hip ratio indicating greater central adiposity (Table 2). There were no significant differences in mean dietary glutamic acid and mean vegetable protein, but the mean animal protein consumed was significantly higher in the obese group (~58 grams) compared to the non—obese group (~49 grams). There were no differences in BDI scores or antidepressant use between the two groups.

**Table 1:** Baseline subjects characteristics.

**Baseline characteristics (*****N*** **= 168) [Mean ± Standard Deviation]**	
Mean age (years) (range)	45.4 ± 11.9 (19–71)
Female (%)	76 (45.2)
Mean body mass index (BMI) (kg/m^2^) (range)	33.5 ± 8.7 (20–58)
Waist/hip ratio (range)	0.96 ± 0.08 (0.78–1.17)
White/caucasian (%)	89 (52.9)
Black/african american (%)	74 (44.3)
Total beck depression inventory (BDI)	14.5 ± 10.2 (2–50)
Diabetes (%)	38 (22.6)
Hyperlipidemia (%)	66 (39)
Hypertension (%)	66 (39)
Current antidepressant use (%)	118 (70)
Chlorpromazine (CPZ) equivalent (mg)	642.2 ± 1249.7 (180–2,000)
Energy (kcal/day) (range)	2,035 ± 763 (1,918–4,793)
Glutamic acid intake (grams/day) (range)	15.5 ± 6.7 (6.06–40.9)
Vegetable protein intake (grams/day) (range)	25.5 ± 11.9 (5.9–61.5)
Animal protein intake (grams/day) (range)	54.4 ± 27.7 (18.1–199.2)

**Table 2:** Subject demographics stratified by obesity.

	**Non-obese (<30 kg/m^**2**^) (*N* = 60)**	**Obese (≥30 kg/m^**2**^) (*N* = 108)**	***P*-value**
**Demographics (means ± standard deviation)**			
Age in years	42.8 ± 12.5 (19–70)	46.7 ± 11.2 (22–71)	0.038
Total number of female subjects (%)	21 (35)	53 (49)	0.045
Total number of white subjects (%)	21 (35.5)	53 (49)	0.32
Total beck depression inventory (BDI)	13.2 ± 10.6 (2–43)	15.2 ± 10.0 (3–50)	0.23
**Comorbid conditions**			
Waist/hip ratio	0.92 ± 0.08 (0.78–1.14)	0.98 ± 0.08 (0.83–1.17)	<0.0001
Diabetes (%)	10 (16.7)	28 (25.9)	0.129
Hyperlipidemia (%)	16 (26.7)	50 (46.3)	0.023
Hypertension (%)	14 (23.3)	52 (48.2)	0.001
**Medications**			
Chlorpromazine (CPZ) equivalents (mg)	548 ± 618 (200–1,800)	695 ± 1,483 (180–2,000)	0.473
Current antidepressant use (%)	38 (63.3)	81 (74.8)	0.12
**Dietary parameters**			
Total caloric intake (kcals/day) (range)	2,062 ± 749 (1,096–4,793)	2,027 ± 791 (1,110–4,436)	0.70
Glutamic acid intake (grams/day)	14.30 ± 4.97 (6.75–25.4)	16.14 ± 7.37 (7.8–37.8)	0.086
Vegetable protein intake (grams/day)	24.79 ± 11.14 (16–54.7)	25.84 ± 12.29 (12–58.4)	0.583
Animal protein intake (grams/day)	48.56 ± 20.48 (25–126)	57.61461 ± 30.63 (23–199)	0.042

Overall, when examining the entire subject group, glutamic acid was not associated with BDI scores after controlling for age, sex, race, antidepressant use, CPZ equivalents in normal control populations or other sources of dietary glutamic acid (i.e., vegetable and animal protein sources) (*p* = 0.42). However, in the models stratified by obesity status, higher glutamic acid level was correlated with higher BDI scores after controlling for animal and vegetable protein sources of glutamic acid (*B* = 2.389, s.e. = 1.04, *p* = 0.021), whereas this was not found for the obese subjects (*B* = −0.438, s.e. = 0.65, *p* = 0.662). For both of these analyses, age, race, sex, CPZ equivalents and antidepressant use, were controlled for in an effort to reduce variability. Each of these analysis had ~a β = 0.7.

## Discussion

Overall, the results of this investigation showed a correlation between greater dietary intake of glutamic acid and increased depressive symptoms among non—obese participants with a schizophrenia spectrum diagnosis. Of note, the participants' level of depressive symptoms, indicating mild mood disturbances, was similar between the two groups, as was current use of antidepressants. Regardless, these results add to the growing evidence regarding the role of dietary additives such as MSG within the field of psychiatry, food additives constitute the highest source of glutamic acid following food, and vegetable protein sources (36). To our knowledge, this is the first investigation to examine the relationships between dietary intake of glutamic acid and depressive symptoms within patients with psychosis, stratified by obesity status.

In examining our subjects' diets, mean glutamic acid consumption was 15.5 grams (range 6–41) which translates in to ~0.2 grams/kg. This is significantly higher than the average of 12 grams/day, commonly seen in a general westernized diet, which is ~0.15 grams/kg for an 80 kg person (37, 38). Physiologically, serum glutamate concentrations have been shown to increase by 250–500% compared to baseline after bolus administration and these elevations are maintained for sustained periods of time, although this is dose dependent (39, 40). Therefore, sustained glutamate serum concentrations due to continuous administration during meals, may be a contributing factor to the occurrence of co-morbid conditions such as obesity and/or depressive disorders. However, it is often difficult to extrapolate results between different subject groups, which necessitates investigations specific to patients with mental illness.

Currently, most data regarding the neuropsychiatric effects of MSG consumption has focused on the occurrence of pain in patients with fibromyalgia or temporomandibular disorders (27, 28, 41). However, while pain did increase in these subjects compared to controls, MSG administration also resulted in more depressive symptomology, specifically greater difficulty concentrating (60 vs. 39%), problems sleeping (83 vs. 53%), impaired attention span (48 vs. 43%), or overall brain fog (61 vs. 48%) (28). Thus, the clinical adverse effects seen may have occurred due to the high risk for psychiatric co-morbidity associated with their neuropsychiatric conditions, similar to our patients with their schizophrenia spectrum diagnosis. Thus, while dietary MSG consumption in general is associated with no or few adverse effects, some patients populations may be at greater risk for significant adverse effects, or may be unknowingly consuming higher doses of glutamic acid which may contribute to these adverse effects (20).

Mechanistically, dietary glutamic acid may be exerting these clinical effects through serotonergic and HPA dysfunction, as animal research has found significantly higher serum levels of cortisol and ACTH, and increased 5-HT uptake in cerebral cortices of rats following MSG administration compared to placebo (21, 42, 43). Additionally, these effects in animals have been found to be reversed by 7,8-dihydroxyflavone, a brain-derived neurotrophic factor/tropomyosin receptor kinase B (BDNF/TrkB) agonist, which mechanistically may reverse glucocorticoid receptor phosphorylation (44). This finding in particular is important to this investigation, as BDNF hypermethylation has been previously reported as a link between obesity and depression in addition to its role in schizophrenia pathophysiology (10, 12, 45). Taken together then, these data suggest that the both the metabolic and behavioral effects of MSG may occur through epigenomic modulation potentially impairing the hypothalamic-pituitary-axis (HPA) feedback inhibition.

Therefore, the results of our study add to the literature regarding the role of dietary glutamic acid concentrations and depressive symptoms in patients diagnosed with schizophrenia spectrum disorder. Although overall, we did not find a relationship between dietary glutamic acid and depressive symptoms overall, we did find differences in the non—obese subject group. The effect seen in this group may be due to fewer cardiovascular risk factors contributing to depression in these individuals, resulting in a greater impact regarding dietary glutamic acid consumption. Additionally, as previously discussed, the role of co-occurring conditions such as obesity, which potentially influences differing inflammatory cascades due to epigenomic modulations, may have also impacted this relationship and mask any correlations between glutamic acid consumption and depressive symptomatology in our obese subjects (46, 47).

Regardless, these relationships should be considered preliminary as future research needs to directly examine the role diet and in particular, MSG consumption on not only depressive symptomatology, and psychotic symptoms in patients with a schizophrenia spectrum diagnosis, but also epigenomic mechanisms behind these potential relationships.

## Limitations

Given the exploratory nature of this study, a few limitations to this work need to be addressed. First, glutamic acid was used as a surrogate measurement for MSG since we were unable to directly measure MSG intake as part of this study. Currently, the Food and Drug Administration (FDA) requires that foods containing MSG note monosodium glutamate as part of their ingredient panel, but it is not currently necessary to quantify the amount of MSG included (21). This limitation is not unique to this investigation, and speaks to the need to continue research examining the relationship between dietary glutamic acid consumption and health effects. In order to overcome this limitation, many studies will place subjects on controlled MSG free diets, or give subjects supplementary doses of MSG. However, for individuals with mental illness, these interventions may be associated with an increased risk of symptom relapse and therefore might not be appropriate. Furthermore, although this study did not measure serum glutamate levels, previous work shows that MSG administration in humans leads to variable changes in serum glutamate concentrations due to unknown reasons (48). Thus, this variability, and our lack of glutamate serum concentrations may also partly explain why our primary significant finding were only seen in the non-obese group, as the mg/kg “dose” of MSG consumed in the obese group would have been smaller. Thus, future work will need to take this under consideration as we examine the relationship between MSG consumption, serum glutamate, and psychiatric symptomatology. We also need to acknowledge that the use of a 24 h food recount in patients with schizophrenia spectrum disorders may be a limitation due to memory recall issues. Lastly, the lack of a control group in the analysis, as well as the small sample size, and in particular the small number of subjects within the non-obese group, are also limitations of this work.

Despite these limitations, this study also exhibited a few strengths, such as the use of the 24 h food frequency questionnaire given on three separate occasions, as this dietary recall data is considered the gold standard and the same methodology used as part of the National Health and Nutritional Examination Study (NHANES) (49). Previous work examining diet has used dietary diaries or 1 day of diet data, resulting in limited validity, particularly among patients with psychotic, and mood disorders who may have memory deficits (50–52).

## Conclusion

In summary, this current study found a correlation between greater dietary intake of glutamic acid and greater depressive symptoms in non-obese adults with a schizophrenia spectrum diagnosis. In contrast, there was no correlation between dietary glutamic acid and depressive symptomology in obese participants. These preliminary observational findings warrant replication and add to the growing evidence on the role of environmental factors, such as diet modifications, as adjunctive non-pharmacological approach for reducing psychiatric symptoms in serious mental illness.

## Data Availability Statement

The data analyzed in this study is subject to the following licenses/restrictions: contain protected heath information. Requests to access these datasets should be directed to Vicki Ellingrod, vellingr@med.umich.edu.

## Ethics Statement

The studies involving human participants were reviewed and approved by University of Michigan IRBMED. The patients/participants provided their written informed consent to participate in this study.

## Author Contributions

PK, AK, and VE: study design, data collection and analysis, and manuscript preparation. AP and AR: data analysis and manuscript preparation. All authors contributed to the article and approved the submitted version.

## Conflict of Interest

The authors declare that the research was conducted in the absence of any commercial or financial relationships that could be construed as a potential conflict of interest.
